# Supplementary material for: Splice-Junction-Based Mapping of Alternative Isoforms in the Human Proteome
Source: Cell Rep. Author manuscript; Available in PMC 2020 Jan 15. (PMC6961840; doi:10.1016/j.celrep.2019.11.026)
Supplement: 2 [file NIHMS1546469-supplement-2.zip › DF1/PXD010154/Lung/Lung_47_HSF4_GKGASAPRGPGMPNSTEPGPSPVPAFLGK.pdf]

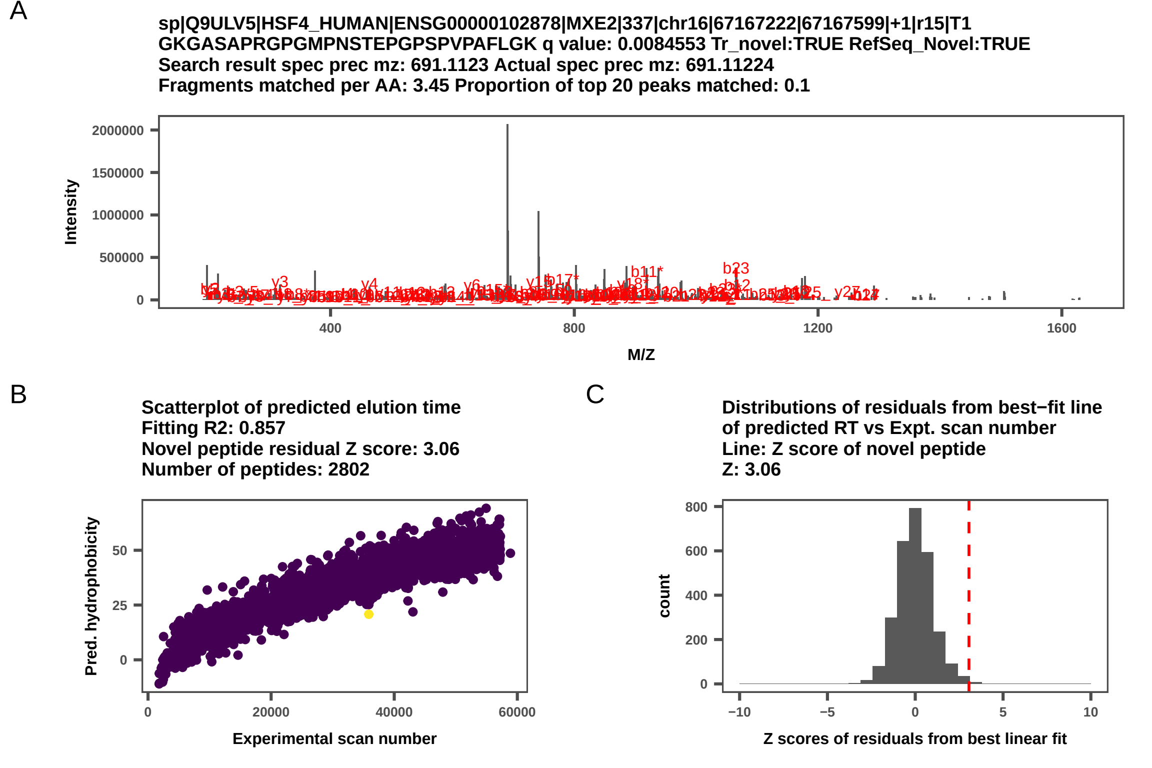
A
sp|Q9ULV5|HSF4_HUMAN|ENSG00000102878|MXE2|337|chr16|67167222|67167599|+1|r15|T1
GKGASAPRGPGMPNSTEPGPSPVPAFLGK q value: 0.0084553 Tr_novel:TRUE RefSeq_Novel:TRUE
Search result spec prec mz: 691.1123 Actual spec prec mz: 691.11224
Fragments matched per AA: 3.45 Proportion of top 20 peaks matched: 0.1
Intensity
2000000
1500000
1000000
500000
0
400
800
1200
1600
M/Z
b23
b11*
y3
y4
y6
b17*
B
Scatterplot of predicted elution time
Fitting R2: 0.857
Novel peptide residual Z score: 3.06
Number of peptides: 2802
Pred. hydrophobicity
50
25
0
0
20000
40000
60000
Experimental scan number
C
Distributions of residuals from best−fit line
of predicted RT vs Expt. scan number
Line: Z score of novel peptide
Z: 3.06
count
800
600
400
200
0
−10
−5
0
5
10
Z scores of residuals from best linear fit
